# 中国滤泡性淋巴瘤诊断与治疗指南（2023年版）

**DOI:** 10.3760/cma.j.issn.0253-2727.2023.07.001

**Published:** 2023-07

**Authors:** 中国抗癌协会血液肿瘤专业委员会 中华医学会血液学分会淋巴细胞疾病学组 中国滤泡淋巴瘤工作组 中国老年保健协会淋巴瘤专业委员会 中国抗癌协会淋巴瘤专业委员会

近年来，滤泡性淋巴瘤（follicular lymphoma，FL）在病理诊断及治疗等方面取得较大进展。为此，中国抗癌协会血液肿瘤专业委员会、中华医学会血液学分会淋巴细胞疾病学组、中国滤泡淋巴瘤工作组（cwFL）、中国老年保健协会淋巴瘤专业委员会和中国抗癌协会淋巴瘤专业委员会组织相关专家，对《中国滤泡性淋巴瘤诊断与治疗指南（2020年版）》进行了修订[1]，制订了本版指南。

一、概述

FL是一类起源于滤泡中心B细胞的非霍奇金淋巴瘤（NHL），典型免疫表型为CD5^−^CD10^+^CD19^+^，伴t（14;18）（q32;q21），临床呈高度异质性。我国FL的发病率占B细胞NHL的8％～23％，低于欧美地区。cwFL分析全国多中心资料，FL诊断时中位年龄约53岁，女性发病率略高于男性，5年的无进展生存（PFS）率及总生存（OS）率分别为61％和89％[2]。

二、病理诊断

（一）取材要点

FL的诊断必须依靠组织病理学检查。FL生物学异质性高，足量、合格的标本组织是确诊、分级以及评估肿瘤生物学特征的重要保证。优先推荐淋巴结/结外病灶的切除/切取活检。对于深部或腔道器官病变，空芯针穿刺或经内镜活检也是可行方式，但要确保有足量标本组织。细针穿刺活检不常规推荐。

（二）组织形态

FL的组织学特征是淋巴组织正常结构被破坏，代之以紧密排列、大小和形状相对单一的肿瘤性滤泡，常累及整个淋巴结并浸润至被膜外，伴或不伴局部弥漫性生长。根据滤泡和弥漫成分所占比例不同可以将FL分为：①滤泡为主型（滤泡比例>75％）；②滤泡-弥漫型（滤泡比例25％～75％）；③局灶滤泡型（滤泡比例<25％）。需要指出的是，如果弥漫区域大细胞成分（中心母细胞或免疫母细胞）较多［每个高倍镜视野（HPF）内>15个］，应单独诊断弥漫大B细胞淋巴瘤（DLBCL）。

根据2022年国际临床咨询委员会公布的成熟淋巴细胞肿瘤分类[3]，FL分级根据滤泡区中心母细胞数量分为1级、2级、3A级及3B级。1级：中心母细胞0～5个/HPF；2级：中心母细胞6～15个/HPF；3级：中心母细胞>15个/HPF，其中，仍有一定数量中心细胞者为3A级，中心细胞罕见者为3B级。1～2级合称为低级别FL。

2022年第5版《WHO造血及淋巴肿瘤分类》对FL的分级提出了新建议[4]，不再强求一定要按1～3B分级，而是将FL分为经典型FL（classic FL, cFL）、滤泡性大B细胞淋巴瘤（follicular large B-cell lymphoma, FLBL）及具有少见特征的FL（FL with uncommon features, uFL）。FLBL取代了原来的FL 3B名称，以强调这组FL和cFL不同的生物学特点。uFL包括“母细胞样”或“大中心细胞”细胞形态和弥漫性生长方式为主型FL，前者更频繁地表现出免疫表型的变异和基因型的多样性，预后较差；后者常表现为腹股沟区大包块，常伴有CD23表达、STAT6突变及1p36缺失或TNFRSF14突变，但无BCL2重排[4]。第5版WHO分类更加注重FL的发病机制，对于指导个体化治疗的意义还有待进一步观察。

（三）免疫表型

FL典型的免疫表型为：CD20^+^、CD10^+^、BCL6^+^、BCL2^+^、CD23^−/+^、CD3^−^、CD5^−^、cyclin D1^−^；CD21、CD23等染色能显示滤泡树突细胞网络的存在；此外，还需注意Ki-67或MYC升高的预后相关性。部分病例（特别是3B级）可以出现CD10^−^或BCL2^−^。

（四）分子特点

FL的分子遗传学检测主要是BCL2重排，细胞遗传学或荧光原位杂交（FISH）检测BCL2基因相关断裂或融合、1p36及IRF4重排可以协助诊断和鉴别诊断。有条件的单位可考虑行二代测序，为FL的精准化诊疗提供依据。

（五）特殊亚型

2022版《WHO造血及淋巴肿瘤分类》还列举了三种特殊FL亚型：

1. 原位滤泡B细胞肿瘤（in situ follicular B-cell neoplasm, ISFN）：通常淋巴结或淋巴组织结构形态无明显异常，但滤泡生发中心内部分B细胞发生t（14;18）（q32;q21）易位，导致BCL2过表达。ISFN需与FL累及部分淋巴结鉴别。少数ISFN有进展为普通FL或其他类型淋巴瘤的风险，需要随访。

2. 儿童型FL（paediatric-type follicular lymphoma, PTFL）：多见于儿童、青少年。多表现为头颈区、偶为腹股沟区等部位孤立性淋巴结肿大，形态学多表现为高级别FL，但遗传学特征与cFL不同，多无BCL2、BCL6和IRF4重排。局部治疗（如手术切除）即可治愈，预后良好。

3. 十二指肠型FL（duodenal-type follicular lymphoma, DTFL）：以侵犯肠道为特征，多局限于小肠，尤多见于十二指肠降部，其形态、免疫表型与低级别FL一致，也有BCL2重排，但遗传特征和cFL不尽相同。通常仅累及黏膜层，少有肠外淋巴结受累。临床表现为惰性，预后较好。

三、分期及预后评估

分期的目的是评估肿瘤的范围及负荷，为治疗提供决策依据。分期体现在初诊时、启动治疗时、疾病进展及再治疗等多个环节。近年来，正电子发射计算机断层显像（PET/CT）逐渐普遍应用于FL的诊疗中。以下情形优先推荐行PET/CT：①诊断为局限期FL，拟采取局部放疗时；②评估治疗结束是否获得完全代谢缓解（CMR）；③临床怀疑发生转化时，协助指导取材部位。FL的分期标准按照2014年版Lugano分期系统[5]。

目前广泛使用的预后评估系统包括FLIPI（Follicular Lymphoma International Prognosis Index）-1和FLIPI-2，具体评分系统见《中国滤泡性淋巴瘤诊断与治疗指南（2020年版）》[1]。近年，临床-生物预后模型，如m7-FLIPI、POD24-PI及23基因积分模型也显示出对预后的良好预测价值，但目前尚未成熟。

四、治疗方案

适用于1～3A级FL，3B级FL的治疗同DLBCL。

（一）治疗前评估

治疗前常规检查同2020年版指南[1]。建议进行肿瘤细胞FISH检查BCL2/IGH易位，对于SUV高代谢部位建议进行活检，必要时可进行超声心动图、尿酸、血清蛋白电泳和（或）免疫球蛋白定量、丙型肝炎相关检测。

（二）治疗原则

FL总体治疗原则是根据分期进行分层治疗，治疗流程见图1。

**图1:**
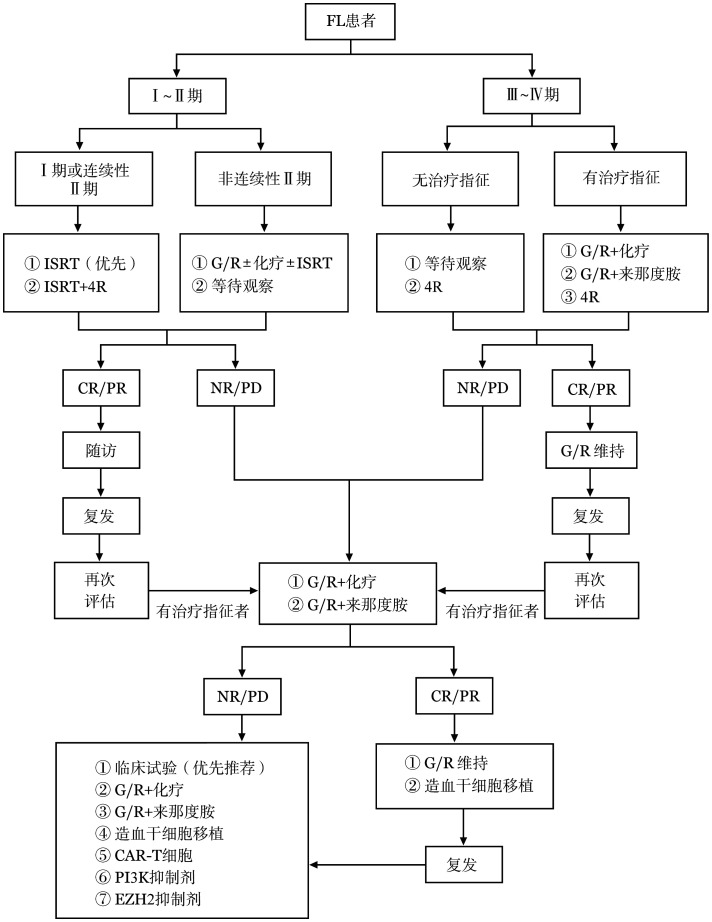
滤泡性淋巴瘤（FL）患者治疗流程图 **注** ISRT：受累部位放疗；G：奥妥珠单抗；R：利妥昔单抗；CR：完全缓解；PR：部分缓解；NR：未缓解；PD：疾病进展；CAR-T细胞：嵌合抗原受体T细胞；PI3K：磷脂酰肌醇3-激酶；EZH2：果蝇Zeste基因增强子人类同源物2

1. Ⅰ～Ⅱ期FL患者的一线治疗选择：Ⅰ～Ⅱ期FL应以积极治疗为主，患者有望得到长期疾病控制。受累部位放疗（involved site radiation therapy，ISRT）是Ⅰ期和连续Ⅱ期患者的标准治疗，推荐放疗剂量为24 Gy，分12次。放疗结束后6个月内进行PET/CT评估，约86％的患者可获得CMR，5年PFS率为69％。对于一些特殊部位（如眼眶等），考虑到放疗相关不良反应，推荐放疗剂量为4 Gy，分2次。远处复发是局限期患者ISRT失败的主要原因，因此治疗前建议行PET/CT分期。对于治疗前无条件行PET/CT检查的患者，建议ISRT后予利妥昔单抗每周1次，共4次治疗。

对于有巨大肿块（≥7 cm）的Ⅰ～Ⅱ期和非连续性Ⅱ期患者可选择抗CD20单抗±化疗±放疗。对于不适合放疗的特殊部位（如腹膜后或肠系膜淋巴结）FL，可考虑抗CD20单抗±化疗。对于完全手术切除的Ⅰ期和不耐受系统治疗或ISRT不良反应的Ⅰ～Ⅱ期患者，也可选择等待观察。

2. Ⅲ～Ⅳ期FL患者的一线治疗：与Ⅰ～Ⅱ期不同，Ⅲ～Ⅳ期FL目前仍被认为不可治愈。因此，无治疗指征者，推荐观察等待；有治疗指征者方可启动治疗，启动治疗的标准参照《中国滤泡性淋巴瘤诊断与治疗指南（2020年版）》[1]。治疗总原则是尽可能延长PFS和OS时间，改善生活质量，同时减少治疗相关不良反应。

对于诊断时无治疗指征的患者，目前优先推荐采取观察等待的策略。既往研究显示，在接受观察等待的患者中，3年和10年时仍无需启动治疗的比例约为46％和20％，中位启动治疗的时间约为31个月。对于部分有强烈治疗意愿的患者，利妥昔单抗每周1次，共4次可作为次要选择，可以显著延长PFS时间和需要再次治疗的时间。

对于诊断时有治疗指征的患者，优先推荐抗CD20单抗+化疗。抗CD20单抗可选择奥妥珠单抗（G）或利妥昔单抗（R），化疗方案可选择苯达莫司汀/CHOP方案/CVP方案。StiL和BRIGHT研究结果显示，BR方案优于R-CVP/CHOP方案，但感染发生率较高，应注意预防[6]–[7]。PET/CT检查最大标准摄取值（SUV_max_）>13[8]或伴果蝇Zeste基因增强子人类同源物2（enhancer of Zeste homolog 2, EZH2）突变[9]患者接受抗CD20单抗+CHOP方案可能获益更大。

GALLIUM研究对比了奥妥珠单抗联合化疗（G-化疗组）和利妥昔单抗联合化疗（R-化疗组）在初治FL中的疗效。中位随访76.5个月的结果显示，G-化疗组PFS持续获益[10]。对研究人群的次要研究终点分析显示，G-化疗组诱导治疗结束后，微小残留病阴性比例更高（92％对85％，*P*＝0.004），提示G-化疗组可更迅速地清除肿瘤细胞并使疾病获得更深层的缓解，G-化疗组一线接受免疫化疗2年内出现疾病进展（POD24）事件风险较R-化疗组降低46％。

来那度胺联合利妥昔单抗或奥妥珠单抗（R^2^或GL）的无化疗方案疗效与免疫化疗类似，也是FL患者的一线治疗推荐，尤其是对于不耐受或不愿接受化疗的患者，但需注意来那度胺的不良反应。

对于一线采用抗CD20单抗联合CVP/CHOP方案治疗6个疗程后获得部分缓解及以上疗效的FL患者，利妥昔单抗或奥妥珠单抗单药维持治疗可显著改善生存。建议每8周应用奥妥珠单抗（1 000 mg）或利妥昔单抗（375 mg/m^2^）维持治疗1次，持续2年。

3. 年老体弱FL患者的治疗：对于年老虚弱不能耐受联合化疗的患者，一线治疗方案可选用利妥昔单抗单药治疗，并加强支持治疗。RELEVANCE研究提示，老年患者也可从R^2^方案中获益[11]。

4. 复发FL患者的治疗原则：复发患者总的治疗原则是延长PFS时间，尽可能降低治疗相关不良反应，改善生活质量。复发时除需再次评估分期、治疗指征及是否发生转化等疾病因素外，还包括体能状况、合并症及既往治疗效果等。首次复发时的治疗对患者的预后至关重要，因此本共识单独列出。

（1）首次复发：目前尚无完善的复发患者的预后评估模型。POD24的患者，5年OS率仅50％[12]，需重点关注。

发生转化是导致FL患者死亡的主要原因，发生率每年2％～3％。当出现某些临床特征时，如乳酸脱氢酶（LDH）短期内迅速升高、淋巴结快速增大、体能状况恶化、新发B症状、高钙血症及新增的结外病灶等，需警惕疾病发生转化。PET/CT检查高代谢部位活检有助于提高诊断的准确率，优先推荐。

首次复发患者的治疗原则建议采用与一线治疗方案非交叉耐药的药物。一线采用R-CHOP/CVP方案治疗结束6个月以上复发的患者，二线选择BR方案，总反应率（ORR）为82％，中位PFS时间34个月[13]；如患者一线采用R-CHOP/CVP方案治疗6个月内无效或复发，则建议选择奥妥珠单抗联合苯达莫司汀作为诱导治疗，然后序贯奥妥珠单抗维持治疗，中位PFS时间为26个月[14]。一线采用BR方案治疗复发的患者，则建议选择R/G联合CHOP/CVP方案。对于一线采用无化疗方案治疗后复发的患者，回顾性研究显示R/G联合化疗作为二线治疗的中位PFS时间为38个月，建议优先考虑[15]。当然，一线治疗缓解时间超过2年的患者，二线治疗也可重新使用原方案，但要注意药物的剂量限制性毒性。

无化疗方案（如R^2^）作为一线免疫化疗复发患者的二线治疗，中位PFS时间为39.4个月，也可选择[16]。

目前认为，对于一线接受含抗CD20单抗治疗复发的患者，二线治疗获得缓解后序贯抗CD20单抗维持治疗仍可获益。但距离末次接受利妥昔单抗治疗6个月内出现疾病进展的患者建议选择奥妥珠单抗维持。

（2）二次及以上复发：二次及以上复发患者治疗前仍需再次评估，参考首次复发时。治疗总原则：①多次复发的患者鼓励优先参加临床试验；②末次治疗方案疗效维持2年以上的患者，再复发时仍可考虑采用原方案；③难治或短期内进展的患者，优先考虑选择作用机制不同的药物，如小分子化合物和嵌合抗原受体T细胞（CAR-T细胞）治疗等；④≥2次复发且复发间隔时间短者或高FLIPI评分的患者可考虑自体造血干细胞移植（auto-HSCT）；⑤auto-HSCT后复发的年轻患者，且有合适的供者，异基因造血干细胞移植（allo-HSCT）可供选择。

磷脂酰肌醇3-激酶（phosphoinositide 3-kinase, PI3K）抑制剂：对于FL总体疗效良好，国内已获批上市的PI3K抑制剂包括度维利塞（duvelisib，PI3Kγ/δ抑制剂）和林普利塞（linperlisib，PI3Kδ抑制剂），单药治疗二次及以上复发FL患者的ORR为42％～80％，中位PFS时间为9～13个月；安全性方面需特别注意肺部感染的发生，发生率为15％左右[17]–[18]。

EZH2抑制剂：他泽司他（Tazemetostat）在难治复发EZH2突变阳性患者中的ORR为69％，其中完全缓解（CR）率为13％，中位缓解持续时间（mDOR）为10.9个月，中位PFS时间为14个月；他泽司他在EZH2野生型患者中的ORR为35％，CR率为4％，mDOR为13个月，中位PFS时间为11个月[19]。

CAR-T细胞治疗：ZUMA-5临床试验显示，既往已接受过2种及以上治疗复发的FL患者接受单次CAR-T细胞治疗的ORR为94％，CR率为79％，随访18个月时，PFS率和OS率分别为67％和87％。整体安全性可控，3级以上细胞因子释放综合征（cytokine release syndrome, CRS）和免疫效应细胞相关神经毒性综合征（immune effector cell-associated neurotoxicity syndrome, ICANS）发生率分别为6％和15％[20]。靶向CD19的CAR-T细胞免疫治疗产品瑞基奥仑赛注射液是国内首个获批用于二线或以上系统性治疗后复发FL的CAR-T细胞产品，其最佳CR率和ORR分别为93％及100％；≥3级CRS和ICANS发生率分别为0及4％[21]。

5. 转化性FL患者的治疗：转化性FL患者治疗方案选择主要考虑的因素包括转化后的病理类型、既往治疗史及患者体能状况等。因转化为高级别B细胞淋巴瘤伴MYC和BCL2重排的比例较高且预后较差，需重点关注并进行相关检查[22]。既往未接受过免疫化疗转化为DLBCL的患者参照初治DLBCL进行治疗，仍可获得较好转归[23]。既往已接受免疫化疗的患者预后相对较差，可供选择的治疗方案有：①参加新药临床试验；②如果化疗敏感，建议采用DLBCL的二线挽救方案，再次缓解后应积极考虑行auto-HSCT巩固；③已有多款CAR-T细胞产品获批上市，ORR约80％，CR率40％～58％，中位PFS时间约11个月，OS时间超过2年[24]–[25]。

6. 造血干细胞移植：因未带来生存获益，FL患者获得首次缓解后不常规推荐行auto-HSCT巩固治疗[26]。接受免疫化疗后POD24的患者再次获得缓解后推荐行auto-HSCT巩固治疗[27]。尽管目前治疗难治复发FL的新药层出不穷，但考虑到治疗相关不良反应、生活质量改善及经济费用等因素，对于年龄小于65岁、二线以上治疗复发的化疗敏感患者，建议考虑行auto-HSCT[28]。allo-HSCT在难治复发FL中的地位尚不明确，有经验的单位可针对性开展研究。

五、疗效评价及随访

参照《中国滤泡性淋巴瘤诊断与治疗指南（2020年版）》[1]。

六、推荐的治疗方案

参照《中国滤泡性淋巴瘤诊断与治疗指南（2020年版）》[1]。
